# Surgeons and Nurses: A Change in the Former's Behaviour

**Published:** 1914-08-01

**Authors:** 


### SURGEONS AND NURSES.

By A FELLOW OF THE ROYAL COLLEGE OF SURGEONS.

The adoption of antisepsis, and subsequently of asepsis, necessitated a very great advance in the training and education of nurses, and was simultaneous with the adoption of the nursing profession by educated ladies in the place of the Sairey Gamps and Betsy Prigs of the pre-Listerian era. Many of these women were as shrewd, as gentle, as conscientious, and in their way as skilled, as the best nurses of the present day; but they were of a very inferior social class, and though many of them performed admirably the duties that were expected of them, these duties were of a simple character, such as could be picked up by an intelligent woman by mere practice in the wards without special teaching or training; and few of the nurses of those days would have been capable of acquiring the high technical skill of the present-day nurse if it had been required of them. Nursing is now advanced to the dignity of a profession. It is true that we have heard a parvenue hostess apologise to her guests for asking them to sit down to table with a nurse, but this was an exposure, not of the inferior status of the nurse, but of the vulgarity and ignorance of the hostess. Educated and cultured ladies of gentle and of aristocratic birth, even princesses of royal houses, now see no degradation in learning the duties of a sick nurse, and in adopting nursing as a calling.

It is the more remarkable, therefore, that the treatment of nurses by some surgeons has undergone at the same time a change in the direction the very reverse of what might have been anticipated. Now that surgeons and nurses belong to the same social class, and that a surgeon may at any time meet in society, and at the table of common friends, the nurses who have had care of his patients and may have care of them again; now that marriages between surgeons and nurses take place every month, and are taken as a matter of course, there are some surgeons who treat nurses in much the same way as a ship's captain treated his crew a hundred years ago, a way in which no ship's captain would venture to treat his crew in these days. The pre-Listerian surgeons—men like Paget, Ferguson, Savory, Hutchinson, Timothy Holmes, Henry Smith, and their colleagues—had to deal with nurses who were scarcely, if at all, above the servant class; and these surgeons treated their nurses with the courtesy with which well-bred gentlemen treat their servants. The present writer, who has witnessed innumerable operations by these great surgeons, never heard so much as an expression of impatience from any one of them towards any of their assistants—anæsthetist, dresser, or nurse. They were gentlemen, and they did not assume that the performance of an operation entitled them to cease to be gentlemen.

There are now surgeons, some of them leading men in large practice and in the highest rank of their profession, who do assume this license. They are either so agitated by the performance of an operation that they lose all self-control; or their gentlemanly feeling is so skin-deep that the slightest breeze of emotion sweeps it all away, and leaves the native boor unconcealed; or they become so inflated with self-importance that they consider the ordinary obligations of courtesy no longer apply to them.

Whatever the explanation, it is notorious that there are surgeons who never address their nurses during an operation without an oath, and rarely address a nurse at any time without a profusion of expletives. Nor is it only in their language that they treat their nurses with contumely and insolence. The following instance appears incredible, and we should not believe it were it not that we have it on the independent words of different nurses, in different employment, entirely unconnected with one another and unknown to each other. A well-known surgeon after an operation was approached by a nurse with a basin of water to wash his hands. The water was not, it appears, of exactly the correct temperature to satisfy the surgeon. It was a trifle too hot or too cool, and therefore he expressed his disapprobation by tilting up the basin towards the nurse, and emptying the whole of its contents over her person. She was drenched to the skin, and had to change her clothing, and, what was of importance to her slender purse, to pay for the washing and ironing of her dress and apron. In our opinion, she would have been justified in breaking the basin on his head, but the poor girl had her living to get, and could not afford to show her resentment. We do not know whether this is a regular or habitual practice on the part of the surgeon in question, but it has been related to us as having occurred more than once, and to different nurses; and the curious part of it is that the surgeon by whom this was done is said to be opposed to the forcible feeding of suffragettes.

